# Characterization of National Medical Societies’ Accessible Resources to Support Underrepresented Minority and Female Trainees

**DOI:** 10.1001/jamanetworkopen.2022.30243

**Published:** 2022-09-06

**Authors:** Lyndsay A. Kandi, Tyler L. Jarvis, Nellie V. Movtchan, Jacob B. Hammond, Chad M. Teven, Alanna M. Rebecca

**Affiliations:** 1 Division of Plastic and Reconstructive Surgery, Department of Surgery, Mayo Clinic, Phoenix, Arizona; 2 Mayo Clinic Alix School of Medicine, Phoenix, Arizona; 3 Department of Surgery, Mayo Clinic, Phoenix, Arizona; 4 Division of Plastic Surgery, Department of Surgery, Northwestern University Feinberg School of Medicine, Chicago, Illinois

## Abstract

**Importance:**

The lack of racial, ethnic, and gender diversity in medicine has been recognized as problematic, but the question of what medical educators and societies are doing to rectify and promote representation of historically marginalized groups persists.

**Objective:**

To examine what easily accessible resources are offered by medical and surgical societies to support women and individuals in minority groups that are underrepresented in medicine (URiM).

**Design, Setting, and Participants:**

This cross-sectional study evaluated transparent and accessible resources on the webpages of societies recognized by the Council of Medical Specialty Societies. Data collection and analysis were performed from September 1, 2021, to November 1, 2021.

**Main Outcomes and Measures:**

The society websites were searched for official diversity statements, diversity and women task forces or committees, and mentorship and scholarship opportunities for URiM and female trainees. The primary outcome was accessible resources in the form of financial support (scholarships) and mentorship for URiM and female trainees.

**Results:**

Of the 45 societies included in the analysis, 38 (84.4%) have published diversity statements. All but 6 societies (86.7%) have a dedicated diversity task force, committee, or work group. Twenty societies (44.4%) have a committee specifically for women or include women in diversity task force initiatives. Seventeen societies (37.8%) offer minority-specific mentorship, 15 (33.3%) offer scholarships targeted toward URiM trainees, 10 (22.2%) provide gender-specific mentorship, and 8 (17.8%) offer scholarship opportunities for female trainees.

**Conclusions and Relevance:**

Although most of the societies included in this study acknowledge the importance of diversity in medicine, less than half of these societies offer readily accessible scholarships or mentorship opportunities to URiM and female applicants.

## Key Points

**Question:**

What tangible and accessible resources, such as scholarships and mentorship, are offered by national medical societies in the support of underrepresented minority and female trainees?

**Findings:**

In this cross-sectional study of all 45 medical and surgical societies recognized by the Council of Medical Specialty Societies, less than half of these societies offered readily accessible scholarships or mentorship opportunities to minority and female trainees despite 84.4% of societies declaring public support of this initiative.

**Meaning:**

The accessibility of resources related to increasing representation in medical societies appears to be lagging behind published statements of support.

## Teaser

This cross-sectional study examines resources for underrepresented minority and female trainees made available by nationally recognized medical and surgical societies.

## Introduction

According to the 2020 US Census data, the overall racial and ethnic diversity of the country has increased since 2010, with the predominant group of non-Hispanic White persons decreasing from 63.7% to 57.8% during a 10-year span.^1^ Despite this shift, medical schools continue to struggle to reflect the diverse population in the US, with proportions of matriculants from racial and ethnic groups underrepresented in medicine (URiM) remaining at levels below their national proportions—an issue that has only worsened during the last 40 years.^2^ Furthermore, research suggests that increasing diversity among health care professionals may lead to culturally competent care as well as improved health care access and outcomes for minority patients.^3,4^ Thus, it is imperative that initiatives to support variation of race and ethnicity within the medical field are undertaken.

Another topic frequently covered under the umbrella of diversity in medicine is gender equity. In recent years, recognition of the disparate gender gap pervasive throughout medicine has driven increased efforts throughout all levels of training—from medical schools to residency and faculty positions—to promote greater gender inclusion.^5,6,7,8,9^ Although women have achieved parity in medical school classes,^10^ work remains to be done in the promotion of more equitable representation in specialties traditionally dominated by men. Indeed, surgical subspecialties continue to lag nonsurgical programs with regard to representation of women,^11^ but ongoing research continues as to why this remains the case despite endeavors to recruit more women.

Change begins first with identification of the issue, thereby allowing an open discussion of potential solutions. However, this lack of diversity in gender as well as ethnic and racial realms has been a reoccurring concern at the national level, with conferences hosted by national organizations, such as the Association of Women Surgeons, and academic journals, such as the *Journal of Racial and Ethnic Health Disparities*, dedicated to these matters. The lack of racial, ethnic, and gender diversity in medicine has been recognized as problematic, but the question of what medical educators and societies are doing to rectify and promote representation of historically marginalized groups persists. To that end, the purpose of this study was to examine what, if any, easily accessible resources are offered to URiM and female trainees by Council of Medical Specialty Societies (CMSS)–recognized medical and surgical societies. We hypothesized that most CMSS-recognized societies (>75%) would offer tangible resources to URiM and female trainees.

## Methods

### Terms

The term *URiM* will be used throughout this manuscript and is defined per the Association of American Medical Colleges (AAMC) as persons of racial and/or ethnic backgrounds who identify as Black, Mexican American, Native American (American Indian, Alaska Native, and Native Hawaiian), and/or mainland Puerto Rican.^12^ The terms *female* and *women* are used interchangeably throughout this article, and they are meant to describe both cisgender and transgender women. *Resources* are henceforth defined as tangible scholarships, mentorship, funding, and networking opportunities. Links to articles on the topic of diversity were not classified as resources. *Easily accessible* is defined as information readily available on websites that did not require email requests for information or membership to access.

### Study Design, Setting, and Participants

This cross-sectional study evaluated transparent and accessible resources on CMSS-recognized medical and surgical societies’ webpages. Data collection and analysis were performed from September 1, 2021, to November 1, 2021. No follow-up was necessary because this study was performed with a cross-sectional design. Institutional review board approval was not required because no patient data were included in this study. This study followed the Strengthening the Reporting of Observational Studies in Epidemiology (STROBE) reporting guideline.

Most societies had options on the drop-down menu of their webpages that redirected the user to their respective diversity, awards and scholarships, and mentorship pages as well as press releases for official societal statements. If a website did not have such selections, the search bar was used for the terms *diversity, diversity statement, committees, female, scholarships,* and *mentorship*. Results of these search terms were investigated for information relating to the data points.

Consecutive sampling of all CMSS-recognized societies was performed, using the CMSS webpage to generate a list of formally recognized medical and surgical societies by.^13^ All 45 societies, including subspecialties, were analyzed.

### End Points

Primary end points include official diversity statements, mentorship, and scholarship opportunities for URiM and female trainees. Secondary end points include diversity and women task forces, committees, or work groups. The Table provides the definitions of these elements.

**Table.** Definition of Diversity and Inclusion Elements Investigated for Each CMSS Society

Element	Definition
Diversity and inclusion statement	A published societal message, separate from the nondiscrimination statement, regarding the importance of equitable representation on the basis of sex, gender, race, and ethnicity
Mentorship opportunities	Mentoring programs geared toward trainees who are considered URiM or female, with qualifications for applying explicitly stating a requirement to identify as one of those groups
Scholarship opportunities	Travel and research grants or other funding opportunities for trainees who are considered URiM or female, with qualifications for applying explicitly stating a requirement to identify as one of those groups
Taskforce, workgroup, or committee	Dedicated group affiliated with the CMSS society that works to draft initiatives and provide resources focused on diversity and/or gender representation

Abbreviations: CMSS, Council of Medical Specialty; URiM, underrepresented in medicine.

### Statistical Analysis 

No advanced statistical analysis was indicated for this study. Sampling bias was controlled for because of the inclusion of all CMSS-recognized societies irrespective of subspecialty.

## Results

### Primary End Points

**Figure 1.**
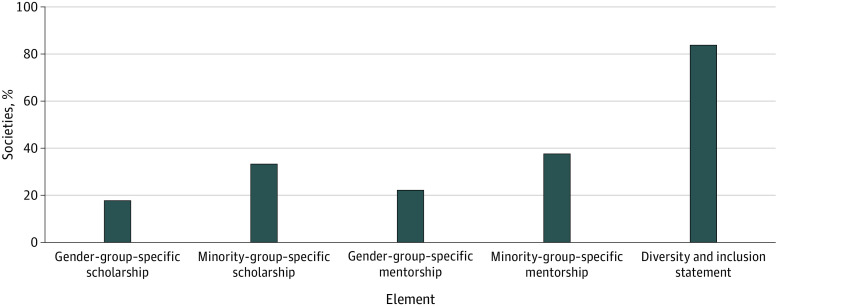
Percentage of the 45 Council of Medical Specialty Societies–Recognized Societies That Display Each Element on Their Website Programs most commonly featured a diversity and inclusion message and minority group–specific resources.

All 45 CMSS-recognized (as of October 27, 2021) medical and surgical societies were included in the analysis (Box). Of the 45 societies, 38 (84.4%) had easily accessible diversity statements published on their respective websites, whereas 7 (15.6%) did not (Figure 1). Regarding tangible resources (Figure 1), 17 societies (37.8%) offered minority-specific mentorship, 15 (33.3%) offered scholarships targeted toward URiM groups, 10 (22.2%) provided gender-specific mentorship, and 8 (17.8%) offered scholarship opportunities for female applicants.

Box. **List of Formally Recognized Societies Included in the Analysis**

**Council of Medical Specialty Societies**

American Academy of Allergy, Asthma and Immunology
American Academy of Dermatology
American Academy of Family Physicians
American Academy of Hospice and Palliative Medicine
American Academy of Neurology
American Academy of Ophthalmology
American Academy of Orthopaedic Surgeons
American Academy of Pediatrics
American Academy of Physical Medicine and Rehabilitation
American Association of Clinical Endocrinology
American College of Cardiology
American College of Emergency Physicians
American College of Medical Genetics and Genomics
American College of Obstetricians and Gynecologists
American College of Occupational and Environmental Medicine
American College of Physicians
American College of Preventive Medicine
American College of Radiology
American College of Rheumatology
American College of Surgeons
American Epilepsy Society
American Gastroenterological Association
American Geriatrics Society
American Medical Informatics Association
American Psychiatric Association
American Society of Anesthesiologists
American Society of Clinical Oncology
American Society for Clinical Pathology
American Society of Colon and Rectal Surgeons
American Society of Hematology
American Society of Nephrology
American Society of Plastic Surgeons
American Society for Radiation Oncology
American Society for Reproductive Medicine
American Thoracic Society
American Urological Association
Infectious Diseases Society of America
North American Spine Society
Society of Critical Care Medicine
Society of Gynecologic Oncology
Society of Hospital Medicine
Society of Interventional Radiology
The Society of Nuclear Medicine and Molecular Imaging
Society of Thoracic Surgeons
Society for Vascular Surgery

List as of September 2021.^13^

### Secondary End Points

**Figure 2.**
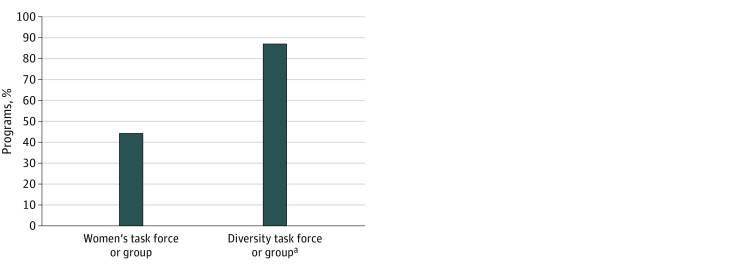
Percentage of the 45 Council of Medical Specialty Societies–Recognized Societies That List Specific Task Force, Committees, or Work Groups on Their Website ^a^Diversity task forces or groups that include gender issues in their initiatives are contained within this label.

Thirty-nine societies (86.7%) had a dedicated diversity task force, committee, or interest group (Figure 2). Twenty societies (44.4%) had a committee, task force, or interest group specifically for women or included gender issues and inclusion of women in their diversity task force initiatives.

## Discussion

During the last 10 years, issues of diversity and inclusion in medicine and surgery have been brought to the forefront of popular discourse throughout the community. Globally, medical schools have been prioritizing the improvement of gender, racial, and ethnic diversity in medical education.^14^ A large and increasing body of evidence suggests that diverse teams consistently outperform homogeneous groups in problem solving, innovation, and the successful completion of other complex tasks.^15,16^ Furthermore, as we learn more about the underpinnings of health disparities across different groups of people, the improvement of diversity in the medical community has become a major topic of discussion.^17^ In light of this growing awareness, we have seen moderate improvement in undergraduate medical education,^18^ but structural racism continues to contribute to differences in achievement of and opportunities for minority students.^19^ Progress in representation of Black^20^ and American Indian and Alaska Native^21^ matriculants have halted if not declined, with the current quota insufficient to serve the representative populations within society.^2^ With a growing, racially diverse population and a lagging relative proportion of minority physicians, there is a dire need to develop sustainable initiatives to increase representation. Although undergraduate medical education institutions for URiM students, such as Howard University, exist, improvement in representation of minority students has been driven by only a handful of schools.^22,23,24,25,26^ Moreover, a bottleneck persists in the pipeline of URiM residents in certain specialties,^26^ largely seen at educational transition points, such as those from medical school graduation to residency.^27^

Overwhelming evidence suggests that high-quality mentorship is key in the pursuit of a career in academic medicine and surgery.^28^ Many URiM students struggle to obtain these relationships. Proposed reasons include the lack of social capital and financial barriers that come with being a first-generation college graduate^29^ as well as the lack of culturally concordant mentors.^28^ Furthermore, URiM students of low socioeconomic status may face financial obstacles in participation in networking events, such as national conferences and meetings. In 2020, the mean number of presentations, abstracts, and publications of seniors at US medical schools who matched to plastic surgery was 19.1.^30^ Often, these works are presented at conferences and meetings, which offer medical students the opportunity to build relationships with potential mentors in the field. With the lack of accessible mentors and the observed proliferation of community-based medical schools focusing on primary and ambulatory care in the last 15 years,^31,32,33^ many URiM students may have limited to no access to a department in their specialty of choice and thus struggle with obtaining the often required letter of recommendation from a department chair. Moreover, many residency programs unofficially require students to rotate at outside institutions during their fourth year for 1-month audition rotations, which are traditionally funded solely by the student. Competitive specialties, such as integrated plastic surgery and orthopedics, often require multiple audition rotations at different institutions to establish ties to particular geographic regions or receive letters of recommendation, enhancing the likelihood of residency interview invitations. The inability to fund these activities can be a significant impediment for URiM students of low socioeconomic status interested in applying for residency training in similarly competitive fields.

Despite the current study demonstrating that less than half of CMSS-recognized medical and surgical societies provide resources for URiM and female trainees, there are several niche societies created to fill this void. In particular, the field of plastic surgery has various institution-specific initiatives and national groups to support the recruitment of these cohorts. An example of this is the Arthur L. Garnes Society, an organization dedicated to “fostering mentorship, collaboration, and fellowship among Black, African-American, and other underrepresented groups in medicine and in plastic and reconstructive surgery.”^34^ In addition, the Women of Color in Plastic and Reconstructive Surgery group has been making rounds on social media as a mentorship opportunity with the aim to “engage, connect, and empower plastic surgeons and plastic surgery trainees who identify as women of color.”^35^ Furthermore, integrated plastic surgery residency programs—often in association with their respective surgery departments—at various institutions, including our own, offer stipends to offset the cost of travel, housing, and fees for visiting fourth-year medical students from diverse backgrounds.

Several studies from various surgical specialties demonstrate the influence of initiatives on career development and recruitment of URiM and female trainees. Specific to orthopedic surgery, the Perry Initiative and Nth Dimensions programs have successful track records in increasing female and URiM representation by offering guidance on career development, clinical exposure, networking opportunities, and research experiences to applicants.^36^ A multifaceted approach devised and implemented at the University of Pennsylvania Health System also increased representation of African American and Latino plastic surgery residents. This monetary travel scholarship and mentorship for URiM students who are pursuing an elective rotation at their institution deemphasizes board scores during residency application review and partners with a group able to perform effective outreach to URiM candidates.^37^ In addition, although the workshop is just beginning, the Plastic Surgery Research, Education, and Preparation Promoting Equity and Diversity Program, funded by the Plastic Surgery Foundation Diversity and Inclusion Grant, aims to prepare medical students from disadvantaged backgrounds for subinternships with the goal of enhancing their success at residency match.^38^ Critics of these initiatives iterate that the data are limited by inclusion bias because students seeking these opportunities may ultimately be in a position of success with or without these programs.

### Limitations

This study has several limitations. Specifically, a formal survey was not used to assess medical societies’ commitment to diversity initiatives. Administering a formal survey might have allowed for more inclusive results, given that some societal websites require membership to access areas of the sites, which entails submission of an application, letter of sponsorship, and fee in most cases. These societies were thereby reported as not readily accessible, although they may have offered scholarships and mentorship to members only; thus, we recognize that these results may not be complete. In the future, a more formal survey of medical societies is warranted to fairly assess meaningful financial outreach and mentorship opportunities. Furthermore, although we focused solely on CMSS-recognized societies, component institutions (societies or programs in association with CMSS) may offer supplemental opportunities. However, we were unsure how to identify component institutions or access potential resources.

## Conclusions

This cross-sectional study found that although most CMSS-recognized medical and surgical societies publish official statements on the importance of diversity and inclusion with respect to recruitment of trainees, fewer than half of these organizations appear to offer scholarships, funding, or mentorship opportunities to support URiM and female applicants. Featuring publicly available resources and initiatives on the CMSS society’s website that display its commitment to equitable racial and gender representation may help to attract a diverse candidate pool. This study serves to highlight the opportunities CMSS societies may offer to demonstrate their commitment to diversity and inclusion.
